# Body Composition, Symptoms, and Survival in Advanced Cancer Patients Referred to a Phase I Service

**DOI:** 10.1371/journal.pone.0029330

**Published:** 2012-01-03

**Authors:** Henrique A. Parsons, Vickie E. Baracos, Navjot Dhillon, David S. Hong, Razelle Kurzrock

**Affiliations:** 1 Department of Investigational Cancer Therapeutics (A Phase I Clinical Trials Program), The University of Texas MD Anderson Cancer Center, Houston, Texas, United States of America; 2 Department of Oncology/Division of Palliative Care Medicine, University of Alberta, Edmonton, Alberta, Canada; Institut Gustave Roussy, France

## Abstract

**Background:**

Body weight and body composition are relevant to the outcomes of cancer and antineoplastic therapy. However, their role in Phase I clinical trial patients is unknown.

**Methods:**

We reviewed symptom burden, body composition, and survival in 104 patients with advanced cancer referred to a Phase I oncology service. Symptom burden was analyzed using the MD Anderson Symptom Assessment Inventory(MDASI); body composition was evaluated utilizing computerized tomography(CT) images. A body mass index (BMI)≥25 kg/m^2^ was considered overweight. Sarcopenia, severe muscle depletion, was assessed using CT-based criteria.

**Results:**

Most patients were overweight (n = 65, 63%); 53 patients were sarcopenic (51%), including 79% of patients with a BMI<25 kg/m^2^ and 34% of those with BMI≥25 kg/m^2^. Sarcopenic patients were older and less frequently African-American. Symptom burden did not differ among patients classified according to BMI and presence of sarcopenia. Median (95% confidence interval) survival (days) varied according to body composition: 215 (71–358) (BMI<25 kg/m^2^; sarcopenic), 271 (99–443) (BMI<25 kg/m^2^; non-sarcopenic), 484 (286–681) (BMI≥25 kg/m^2^; sarcopenic); 501 d (309–693) (BMI≥25 kg/m^2^; non-sarcopenic). Higher muscle index and gastrointestinal cancer diagnosis predicted longer survival in multivariate analysis after controlling for age, gender, performance status, and fat index.

**Conclusions:**

Patients referred to a Phase I clinic had a high frequency of sarcopenia and a BMI≥25 kg/m^2^, independent of symptom burden. Body composition variables were predictive of clinically relevant survival differences, which is potentially important in developing Phase I studies.

## Introduction

Several body composition features have been associated with the incidence, etiology, and therapeutic outcomes of cancer. Obesity, as one example, has been implicated in the etiology and prognosis of various cancers [1]. Additionally, weight loss is frequent among cancer patients, especially in advanced disease [2], and is the predominant feature of cancer cachexia [3]. Cachexia occurs in up to 80% of cancer patients [4], is a marker of poor prognosis [5], [6], [7], negatively impacts patients' quality of life [8], [9], and impairs their normal physical function [10]. Sarcopenia, severe muscle depletion, has received special attention in the recent cancer literature because of its association with reduced physical ability and increased mortality in noncancer patients [11], [12], [13], [14], and unfavorable treatment outcomes, especially severe toxicity [12], [15], [16]. Studies in patients with malignant diseases [16], [17] and non-malignant conditions [18], [19] have shown that the combination of heavy body weight and sarcopenia results in particularly poor physical functional ability and clinical outcome.

Various mechanisms putatively underlie muscle wasting and cachexia. Inflammation is a likely major player in the genesis of these entities, and the relationship between cachexia and cytokines has been widely studied [20], [21], [22], [23], [24]. Inflammatory pathways and cytokines have also been implicated in cancer-related symptoms [25], [26], [27], [28], which cause severe distress and impair the quality of life of cancer patients, especially those with advanced disease. A rational hypothesis is that sarcopenia, cachexia and other cancer-related symptoms share similar underlying inflammatory mechanisms.

The rapidly developing field of oncology has been driven, in part, by clinical trials, reflected by the 5,841 active and recruiting oncology phase I and II studies listed on the www.clinicaltrials.gov website as of early May 2011. Patients enrolled on these trials typically have failed to respond to multiple standard-of-care therapeutic regimens and frequently have less than a one-year expected survival [29]. Ideally, candidates for accrual to these investigations survive long enough to generate meaningful results, and have a minimum of features to confound the interpretation of results (i.e., significant symptom burden, unusual propensity for treatment toxicity). There is a dearth of research examining the relationships among body composition, the incidence and severity of cancer-related symptoms, and survival in patients with advanced cancer. Therefore, we assessed these variables in 104 patients referred to the Phase I clinic at The University of Texas MD Anderson Cancer Center Department of Investigational Therapeutics.

## Methods

A symptom questionnaire was completed by 124 patients with advanced cancer who were referred to the Phase I clinic and who agreed to participate. Patients participating in the study were ≥18-years old with documented advanced cancer. The study was approved by the MD Anderson Institutional Review Board (IRB) and informed consent was obtained from each patient.

### Symptom Inventory

Patients completed the MD Anderson Symptom Inventory (MDASI) [30], a validated questionnaire used to assess the intensity of 15 cancer-related symptoms (pain, fatigue, nausea, sleep, distress, dyspnea, memory, appetite, drowsiness, xerostomia, sadness, vomiting, numbness, coughing, and constipation). The MDASI also assesses how patients' symptoms interfere with six specific life domains (general activity, mood, normal work, ability to walk, interpersonal relations, and enjoyment of life). All symptom items are rated on an 11-point numeric scale from 0 (“no symptom at all”) to 10 (“worst imaginable symptom”). A composite symptom score ranging from 0 to 10 was computed using the sum of all 15 symptom scores divided by 15. Interference in the six life domain items was also rated according to a numeric 11-point scale ranging from 0 (“does not interfere”) to 10 (“completely interferes”), and a composite score was similarly obtained [30].

### Demographic Data

Patient demographic data, including age, gender, ethnicity, cancer diagnosis, height, and weight at the time of presentation to the Phase I clinic were collected by reviewing the electronic medical records of the patients assessed in our analysis. When no information about patient weight was available for specific clinic appointments, the information was obtained from the closest date in the patient's medical chart, which was a median of 5 days before symptom assessment (range, 1–14). Death date was obtained from the chart or from the Social Security Death Index for patients whose medical records did not contain this information [31], [32]. Patients with no verifiable death date were censored at the date of their last follow-up appointment.

### Body Composition Assessments

Body mass index (BMI) was calculated by dividing the patient's weight in kilograms by height (in meters) squared [33]. Lean body mass and muscularity were calculated using the validated method described below.

**Figure 1:**
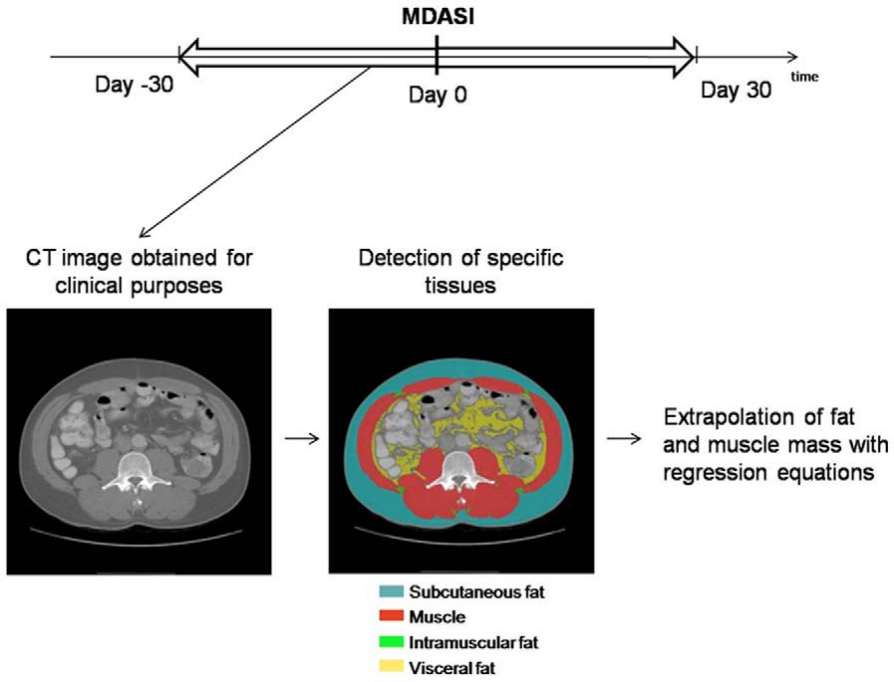
Image Acquisition and Analysis. Computerized Tomography images requested for clinical purposes within 30 days of the completion of the symptom questionnaire (MDASI) were downloaded locally and the different tissues identified at the L3 level. Posteriorly, the cross-sectional areas determined are applied to regression equations to estimate total body fat and muscle compartments.

Computerized tomography (CT) image sets obtained for clinical purposes no more than 30 days before or after the symptom questionnaire was filled out were identified by chart review (median time from image to MDASI, 2 days, interquartile range 1–8 days). Abdominal images at the level of the 3^rd^ lumbar vertebra (L3) were used for body composition analysis. The 3^rd^ lumbar vertebra CT cross-sectional image was chosen for analysis because it contains the following muscles: *psoas*, *erector spinae*, *quadratus lumborum*, *transversus abdominus*, *rectus abdominus*, and the external and internal oblique muscles, which together are optimal for estimating lean body mass. The use of the 3^rd^ lumbar vertebra as the landmark for body composition analysis has been previously described and validated against dual X-ray absorptiometry and bioimpedance analysis in healthy populations and in patients with advanced cancer [34], [35], [36]. Muscles, subcutaneous fat, and visceral fat were identified by a single assessor trained in the specific anatomy of these tissues, demarcated using previously described Hounsfield unit thresholds [37], [38], [39] and quantified with SliceOMatic software, version 4.3 (Tomovision, Montreal, QC, Canada). Whole body composition as well as lean and fat body mass were estimated by applying the values obtained for muscularity (LBM = lean body mass) and adiposity (FM = fat mass) at the L3 level to the Mourtzakis et al. formulae: 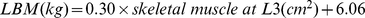 and 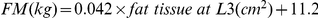 with demonstrated reliability (r = 0.94, p<0.0001 and r = 0.88, p<0.0001, respectively) [34]. Patients were considered to be sarcopenic if they had a lumbar skeletal muscle index (skeletal muscle area at L3 divided by the height squared) lower than 38.5 cm^2^/m^2^ for women and lower than 52.4 cm^2^/m^2^ for men, as previously described [16]. This process is summarized in Figure 1. Fat index was determined by dividing total adipose tissue area at L3 by the height squared. To further investigate relationships among BMI, sarcopenia, symptoms, and survival, we classified patients into 4 groups according to their BMI (<25 kg/m^2^ and ≥25 kg/m^2^) and the presence or absence of sarcopenia.

### Statistical Analyses

Descriptive statistics were used to summarize our data. Differences in categorical variables were determined by chi-square and Fisher's exact tests, when applicable. Differences in continuous variables were determined by t-tests or by the Mann-Whitney test, depending on the normality of the data. Differences in continuous variables across three or more groups were determined by one-way ANOVA. Survival analyses were done using the Kaplan-Meier and Cox Regression methods. Significance level cutoff was 0.05. Analyses were performed using SPSS v. 16.0 computer software (SPSS Inc., Chicago, IL).

## Results

### Anthropometrical and Body Composition Data

From the initial 124 patients who completed the symptom questionnaire, evaluable CT images within 30 days of the completion of the symptom questionnaire were available for 114 patients (92%) and, of those, 10 (∼9%) did not have technically suitable images (eight had part of the subcutaneous adipose tissue image cut because of the original imaging framing and two had extensive surgical procedures changing the usual anatomy of the L3 level images). Of the 104 evaluable patients, 53 were sarcopenic (51%). The difference in rate of sarcopenia did not attain statistical significance in men versus women (55% vs. 44%, p = 0.312). Overall, patients≥65 years were more likely to be sarcopenic (25/35, 71% vs. 28/69, 41%, p = 0.003) and African Americans were less likely to be sarcopenic (1/9, 11% vs. 52/95, 55%, p = 0.015). Sarcopenia was present in 31/38 (79%) of patients with a BMI<25 kg/m^2^ and in 22/65 (34%) of patients with a BMI≥25 kg/m^2^ (p<0.0001). Underweight patients (BMI≤18.5 kg/m^2^) accounted for only approximately 3% of the study population (3 patients). Therefore, they were grouped with the normal weight patients for all analyses.

Body composition and anthropometrical features are reported in Table 1. Significant differences were detected among the four groups (BMI<25 kg/m^2^ non-sarcopenic, BMI<25 kg/m^2^ sarcopenic, BMI≥25 kg/m^2^ non-sarcopenic, and BMI≥25 kg/m^2^ sarcopenic) with regards to all anthropometrical features and body composition.

**Table 1:** Anthropometric and demographic data according to BMI and presence/absence of sarcopenia.

Body mass index	<25 kg/m^2^				≥25 kg/m^2^				
Sarcopenia	No		Yes		No		Yes		P*
	n	SEM(or %)	n	SEM(or %)	n	SEM(or %)	n	SEM(or %)	
**Age** (years)	54.3	2.9	61.0	1.6	56.0	1.9	64.0	1.9	**0.012**
**Gender**									
Female	3	38%	13	42%	19	44%	4	18%	0.205
Male	5	63%	18	58%	24	56%	18	82%	
**Race**									
Caucasian	5	63%	27	87%	32	74%	19	86%	0.062
Hispanic	0	-	2	6%	6	14%	3	14%	
African-American	3	38%	1	3%	5	12%	0	-	
Other	0	-	1	3%	0	-	0	-	
**Diagnosis**									
Gastrointestinal	2	25%	13	42%	12	28%	9	41%	0.825
Head/Neck	3	38%	8	26%	13	30%	7	32%	
Others	3	38%	10	32%	18	42%	6	27%	
**Performance Status**									
0–1	6	75%	29	94%	39	91%	21	96%	0.336
2–3	2	25%	2	6%	4	9%	1	4%	
**Body mass index** (kg/m^2^)	23.2	0.5	22.0	0.4	31.6	0.8	28.6	0.7	**<0.001**
**Skeletal muscle cross-sectional area** (cm^2^)	150.3	13.0	117.3	5.2	154.6	5.1	136.4	6.1	**<0.001**
**Intramuscular adipose cross-sectional area** (cm^2^)	7.5	1.5	8.3	0.8	13.2	1.3	16.1	1.8	**<0.001**
**Visceral adipose cross-sectional area** (cm^2^)	29.0	9.3	66.4	9.7	158.1	10.7	180.5	15.8	**<0.001**
**Subcutaneous adipose cross-sectional area** (cm^2^)	133.4	18.2	130.8	10.9	292.0	18.8	231.8	19.8	**<0.001**
**Estimated total lean body mass** (kg)	51.1	3.9	41.2	1.6	52.4	1.5	47.0	1.8	**<0.001**
**Estimated total fat mass** (kg)	18.3	1.1	19.8	0.7	30.7	0.9	29.2	0.9	**<0.001**
**Lumbar skeletal muscle index (cm^2^/m^2^)**	50.6	2.9	40.3	1.2	54.1	1.3	44.7	1.4	**<0.001**
**Lumbar fat index (cm^2^/m^2^)**	6.3	0.4	7.0	0.3	10.9	0.4	9.7	0.4	**<0.001**

* = chi square p value for the first four categories. Otherwise, ANOVA.

### Body Composition and Survival

Overall median survival (95% confidence interval), assessed from the date when the initial CT image was obtained, was 400 days (range, 270–530). There was a trend towards shorter median survival among sarcopenic compared to non-sarcopenic patients (304 days [range, 201–406] versus 474 days [range, 346–601]), respectively, but the difference did not attain statistical significance (p = 0.151). Patients≤65 years with sarcopenia had significantly shorter survivals compared to patients without sarcopenia (301 versus 487 days, respectively, p = 0.042).

**Figure 2:**
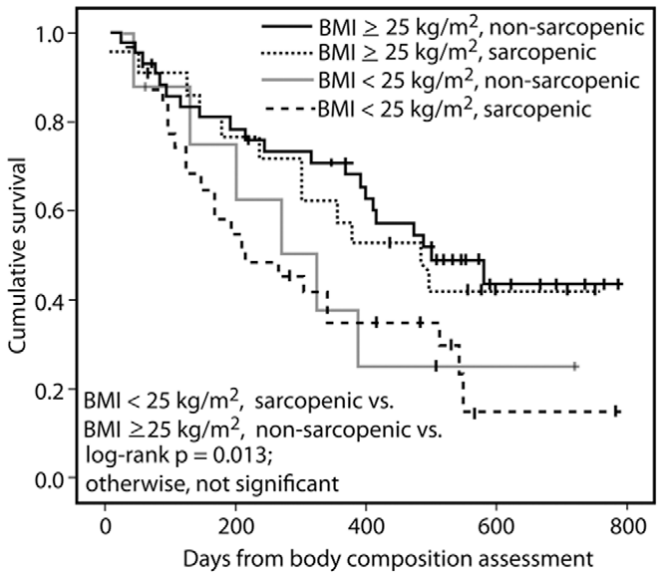
Kaplan-Meier curves of the four groups of patients. Survival analyses were performed using Kaplan-Meier curves and log-rank tests to detect differences in survival among the four groups of patients (normal weight non-sarcopenic, normal weight sarcopenic, overweight non-sarcopenic, and overweight sarcopenic patients. Patients who are alive at last known follow up are censored at that date.

Survival differed across the four groups according to BMI and presence of sarcopenia, as depicted in Figure 2. Patients with a BMI<25 kg/m^2^ with sarcopenia had the shortest survival (median 215 days, 95% confidence interval 99–443 days), whereas patients with a BMI≥25 kg/m^2^ without sarcopenia fared best (median survival 501 days, 95% confidence interval 309–693 days, log-rank p = 0.013).

After controlling for the effects of age, gender, performance status, and fat index in multivariate analysis, patients with higher muscle indices had longer survivals (hazard ratio 0.955, confidence interval 0.923–0.989, p = 0.009), as did those with gastrointestinal malignancies, with a hazard ratio of death of 0.509 (confidence interval 0.307–0.845, p = 0.009) (Table 2).

**Table 2:** Predictors of survival – multivariate analysis.

	Univariate analyses*			Multivariate analysis**		
	Hazard Ratio	CI	p value	Hazard Ratio	CI	p value
Age	1.004	0.982–1.026	0.720	-	-	-
Female gender	0.962	0.568–1.628	0.885	0.710	0.492–1.024	0.067
Performance status	1.514	1.030–2.226	**0.035**	1.458	0.974–2.184	0.067
Diagnosis (GI vs. others)	0.445	0.270–0.735	**0.002**	0.509	0.307–0.845	**0.009**
Muscle Index	0.974	0.951–0.998	**0.036**	0.955	0.923–0.989	**0.009**
Fat index	0.921	0.835–1.015	0.098	-	-	-

*Cox Univariate analyses.

**Backward elimination method of Cox Proportional Hazards Model.

### Symptom Burden

Overall symptom burden is described in Table 3. Fatigue was the most frequently reported symptom (93/104 patients, 90%) and vomiting was the least reported (25/104 patients, 24%).

**Table 3:** MDASI scores – The MDASI includes questions about 15 symptoms frequent in advanced cancer patients.

	BMI<25 kg/m^2^				BMI≥25 kg/m^2^				
Symptoms	Non-sarcopenic		Sarcopenic		Non-sarcopenic		Sarcopenic		ANOVA p
	Mean (SEM)		Mean (SEM)		Mean (SEM)		Mean (SEM)		
Pain	2.6	(0.7)	2.8	(0.5)	2.3	(0.4)	2.2	(0.6)	0.822
Fatigue	4.1	(0.6)	4	(0.5)	3.4	(0.4)	4.3	(0.6)	0.605
Nausea	1.4	(1)	1.8	(0.5)	1.7	(0.4)	1.2	(0.5)	0.843
Insomnia	1.5	(0.8)	2.8	(0.5)	2.3	(0.4)	2.9	(0.6)	0.536
Distress	2.6	(1)	1.5	(0.3)	2.7	(0.4)	2.5	(0.6)	0.208
Dyspnea	2.3	(0.9)	2.1	(0.5)	1.5	(0.3)	1.7	(0.6)	0.696
Memory loss	1.6	(0.5)	2	(0.4)	2	(0.4)	2.8	(0.5)	0.592
Anorexia	1.9	(1.1)	2.8	(0.6)	2	(0.5)	1.7	(0.5)	0.557
Drowsiness	2.9	(0.8)	2.5	(0.5)	2.5	(0.4)	3	(0.5)	0.818
Dry mouth	2.8	(1.3)	2.2	(0.4)	2	(0.4)	3	(0.7)	0.475
Sadness	1.6	(0.9)	1.2	(0.3)	2.1	(0.5)	2	(0.4)	0.458
Vomiting	1.4	(1)	0.9	(0.3)	1.1	(0.4)	0.5	(0.4)	0.685
Numbness	2.3	(1)	2.2	−0.5	1.3	(0.3)	2.2	(0.4)	0.268
Coughing	1.4	(0.8)	1.1	(0.3)	1.8	(0.4)	1.1	(0.5)	0.531
Constipation	1.8	(0.7)	1.6	(0.4)	1.2	(0.3)	2	(0.7)	0.549
Symptom composite	2.1	(0.5)	2.1	(0.2)	2	(0.2)	2.2	(0.3)	0.952

Patients grade their symptoms on a 0–10 scale in which 0 represents “no symptom at all” and 10 represents “worst symptom imaginable”. Mean symptom scores and standard error of the means (SEM) are presented.

No statistically significant differences in symptom burden were found among the four groups according to BMI and presence of sarcopenia. Symptom severity was generally low, with an average composite score of 2.1 (standard error ±0.14). The MDASI interference scores are shown in Table 4. There was a low degree of interference with all six life domains. No statistically significant differences were found among the four groups according to BMI and presence of sarcopenia. However, patients with sarcopenic obesity (sarcopenia and BMI≥30 kg/m^2^) reported greater mean interference scores for mood compared to patients without sarcopenic obesity (mean ± standard error 4.4±1.2 versus 2.1±0.20, respectively, p<0.05).

**Table 4:** MDASI interference scores – The MDASI also includes six questions about the interference of the symptoms on different life domains.

	BMI<25 kg/m^2^				BMI≥25 kg/m^2^				
	Non-sarcopenic		Sarcopenic		Non-sarcopenic		Sarcopenic		p
	Mean (SEM)		Mean (SEM)		Mean (SEM)		Mean (SEM)		
Activity	2.4	(0.6)	3.2	(0.5)	2.4	(0.4)	2.4	(0.6)	0.58
Mood	2.4	(0.7)	2.4	(0.5)	2	(0.4)	2.3	(0.6)	0.897
Working ability	2.3	(0.7)	3.6	(0.6)	2.7	(0.4)	2.5	(0.6)	0.374
Relationships	1.8	(0.8)	2	(0.5)	1.4	(0.3)	1.7	(0.5)	0.835
Walking ability	2	(0.8)	3.2	(0.5)	2.3	(0.4)	2.5	(0.5)	0.489
Enjoyment of life	2.5	(0.7)	3.1	(0.5)	2.1	(0.4)	1.8	(0.5)	0.272
Composite Score	2.2	(0.6)	2.9	(0.5)	2.1	(0.3)	2.2	(0.5)	0.528

Mean interference scores and standard error of the means (SEM) are presented.

## Discussion

When patients are enrolled on clinical trials of investigational agents or regimens, they are monitored closely for side effects, and trial endpoints typically include toxicity and survival assessments [40], [41]. Patients with cancer participating in such trials have usually failed several lines of standard treatment and have advanced disease. Their expected survival is relatively short [29] and they often suffer from diverse symptoms [42]. Cachexia is a frequent complication of cancer, and the interrelationship between symptom burden, body composition, and survival may play a role in how patients tolerate treatment drugs and their outcome. Yet, very little is known about these interrelationships. Previously, other groups have studied distinct potential predictors of clinical outcomes in the Phase I setting. Italiano and collaborators have observed in a sample of 180 patients enrolled into Phase I trials that time between cancer diagnosis and enrollment in the clinical trial greater or equal to 24 months and evidence of treatment response were predictors of greater overall survival [43]. Arkenau and collaborators showed that overall survival of 212 patients enrolled in oncology Phase I trials could be predicted by a score (the Royal Marsden Hospital - RMH score) that included albumin greater than 35 g/L, lactate dehydrogenase greater than the upper limit of normality, and two or more sites of metastases [44]. The RMH score has been independently validated by our group in a sample of 229 patients enrolled in Phase I trials [45]. The current study is a preliminary assessment of the associations among body composition, symptom burden and survival in 104 patients with advanced cancer referred to the Phase I clinic at MD Anderson.

We focused on the body composition aspect of sarcopenia because it has been associated with shorter survival in cancer patients, and seems be a central factor in the genesis of chemotherapy toxicity [15], [16]. We found a 51% frequency of sarcopenia in our patients. This can be compared to a small number of published reports on patients with solid tumors. For instance, Prado et al., in a study of 250 obese patients with respiratory and gastrointestinal cancers, reported that the proportion of sarcopenic patients was 15% [16]. Similarly, we found that 29% of our patients were obese (BMI≥30 kg/m^2^) and 17% (5/30) of our obese patients were sarcopenic. Antoun et al. recently showed in a study of 80 patients with advanced renal cell carcinoma, a frequency of sarcopenia of 72% among patients with a BMI<25 kg/m^2^ and 34% in patients with a BMI≥25 kg/m^2^, similar to our findings of 79% and 34%, respectively [46]. The Prado group reported sarcopenia in 25% of 55 women with metastatic breast cancer receiving capecitabine [15], which is lower than our finding of sarcopenia in 44% of women (17/39). In a comparable sample of 111 patients with advanced pancreatic cancer receiving palliative chemotherapy, Tan et al. observed a 56% prevalence of sarcopenia [17], which is similar to the 51% overall prevalence in our study population.

We also observed a trend towards sarcopenia being more common in older patients consistent with previous reports demonstrating that sarcopenia is more prevalent among the elderly. This relationship is not surprising since muscle loss is a process normally associated with aging [23], [47], [48], [49]. Additionally, we found that African American individuals were less frequently sarcopenic than others. This finding is consistent with previously reported data from a large population (N = 3,000) showing that African Americans had a greater proportion of lean body mass than other racial groups [50].

Overall, our patients reported symptoms that were mild in intensity (mean composite symptom score of ∼2.0), a value similar to that described by Finlay et al. in a Phase I population [42] The relatively low symptom burden may be attributable to the strict eligibility requirements for many early phase clinical trials, leading to referral for clinical trial participation of patients with a good performance status, despite having advanced disease. No statistically significant differences in symptom intensity or interference with function were detected when we compared symptom burden across the four combinations of BMI (cutoff 25 kg/m^2^) and sarcopenia (present/absent).

Overall survival from the time that imaging studies were initially obtained for our patients was ∼13 months. Patients who were overweight (BMI≥25 kg/m^2^) but without sarcopenia fared best, with an approximately 130% longer median survival compared to patients with a BMI<25 kg/m^2^ with sarcopenia (median survival 501 vs. 215 days, respectively). The feature of sarcopenia in lower body weight patients predicts an atypically short survival in patients otherwise meeting the criteria for Phase I trials. Conversely, heavy body weight was associated with a longer median survival. A so-called “obesity paradox” is described, albeit poorly understood, in cardiovascular diseases [51] and renal insufficiency [52], [53], conditions in which patients with higher body mass indices seem to survive longer. In the cancer setting, overweight patients may only apparently survive longer because individuals who are impacted most by their disease lose weight and are classified as having normal weight or even as being underweight, depending on their baseline status. Previous studies have repeatedly showed that sarcopenia has a negative impact on survival. For example, Tan et al. recently demonstrated that sarcopenia is a poor prognostic factor among overweight and obese patients with pancreatic cancer [17]. A similar finding was reported by Prado et al. in a study that included patients with gastrointestinal and respiratory cancers and concurrent obesity [16].

In conclusion, sarcopenia occurred frequently in our patients with advanced cancer referred for clinical trials. Younger age, African American race, and overweight patients (BMI≥25 kg/m^2^) were less likely to be sarcopenic. Although there was a trend for sarcopenic patients to have an increased symptom burden, it did not reach statistical significance. Further, although sarcopenic patients had a six-month shorter average survival than non-sarcopenic patients, this trend was not statistically significant, except in patients less than 65 years old (p = 0.042). Multivariate analysis showed that muscle index was an independent prognostic factor, with patients having greater muscularity faring better. Patients who did the best had a BMI≥25 kg/m^2^ and were non-sarcopenic, and those who fared worst had a BMI<25 kg/m^2^ and were sarcopenic. These data suggest that sarcopenia, weight and other body composition variables merit further study to determine their predictive value in populations of cancer patients being considered for Phase I trial participation and may help better characterize and redefine existing prognostic indices for Phase I settings.

## References

[1] Murthy NS, Mukherjee S, Ray G, Ray A (2009). Dietary factors and cancer chemoprevention: an overview of obesity-related malignancies.. J Postgrad Med.

[2] Sarhill N, Mahmoud F, Walsh D, Nelson KA, Komurcu S (2003). Evaluation of nutritional status in advanced metastatic cancer.. Support Care Cancer.

[3] Evans WJ, Morley JE, Argiles J, Bales C, Baracos V (2008). Cachexia: a new definition.. Clin Nutr.

[4] Ma G, Alexander HR, Bruera E, Portenoy R (1998). Prevalence and pathophysiology of cancer cachexia.. Topics in Palliative Care.

[5] Deans DA, Wigmore SJ, de Beaux AC, Paterson-Brown S, Garden OJ (2007). Clinical prognostic scoring system to aid decision-making in gastro-oesophageal cancer.. Br J Surg.

[6] Fearon KC, Voss AC, Hustead DS (2006). Definition of cancer cachexia: effect of weight loss, reduced food intake, and systemic inflammation on functional status and prognosis.. Am J Clin Nutr.

[7] Dewys WD, Begg C, Lavin PT, Band PR, Bennett JM (1980). Prognostic effect of weight loss prior to chemotherapy in cancer patients. Eastern Cooperative Oncology Group.. Am J Med.

[8] Fouladiun M, Korner U, Gunnebo L, Sixt-Ammilon P, Bosaeus I (2007). Daily physical-rest activities in relation to nutritional state, metabolism, and quality of life in cancer patients with progressive cachexia.. Clin Cancer Res.

[9] Hinsley R, Hughes R (2007). ‘The reflections you get’: an exploration of body image and cachexia.. Int J Palliat Nurs.

[10] Dahele M, Skipworth RJ, Wall L, Voss A, Preston T (2007). Objective physical activity and self-reported quality of life in patients receiving palliative chemotherapy.. J Pain Symptom Manage.

[11] Morley JE, Baumgartner RN, Roubenoff R, Mayer J, Nair KS (2001). Sarcopenia.. J Lab Clin Med.

[12] Roubenoff R (2008). Excess baggage: sarcopenia, obesity, and cancer outcomes.. Lancet Oncol.

[13] Roubenoff R (2002). Exercise, sarcopenia, cognition, and mood.. Nestle Nutr Workshop Ser Clin Perform Programme.

[14] Roubenoff R (2003). Sarcopenia: effects on body composition and function.. J Gerontol A Biol Sci Med Sci.

[15] Prado CM, Baracos VE, McCargar LJ, Reiman T, Mourtzakis M (2009). Sarcopenia as a determinant of chemotherapy toxicity and time to tumor progression in metastatic breast cancer patients receiving capecitabine treatment.. Clin Cancer Res.

[16] Prado CM, Lieffers JR, McCargar LJ, Reiman T, Sawyer MB (2008). Prevalence and clinical implications of sarcopenic obesity in patients with solid tumours of the respiratory and gastrointestinal tracts: a population-based study.. Lancet Oncol.

[17] Tan BH, Birdsell LA, Martin L, Baracos VE, Fearon KC (2009). Sarcopenia in an overweight or obese patient is an adverse prognostic factor in pancreatic cancer.. Clin Cancer Res.

[18] Bouchard DR, Dionne IJ, Brochu M (2009). Sarcopenic/obesity and physical capacity in older men and women: data from the Nutrition as a Determinant of Successful Aging (NuAge)-the Quebec longitudinal Study.. Obesity (Silver Spring).

[19] Rolland Y, Lauwers-Cances V, Cristini C, Abellan van Kan G, Janssen I (2009). Difficulties with physical function associated with obesity, sarcopenia, and sarcopenic-obesity in community-dwelling elderly women: the EPIDOS (EPIDemiologie de l'OSteoporose) Study.. Am J Clin Nutr.

[20] Argiles JM, Busquets S, Lopez-Soriano FJ (2006). Cytokines as mediators and targets for cancer cachexia.. Cancer Treat Res.

[21] Argiles JM, Busquets S, Lopez-Soriano FJ (2003). Cytokines in the pathogenesis of cancer cachexia.. Curr Opin Clin Nutr Metab Care.

[22] Argiles JM, Garcia-Martinez C, Llovera M, Lopez-Soriano FJ (1992). The role of cytokines in muscle wasting: its relation with cancer cachexia.. Med Res Rev.

[23] Jensen GL (2008). Inflammation: roles in aging and sarcopenia.. JPEN J Parenter Enteral Nutr.

[24] Saini A, Al-Shanti N, Stewart CE (2006). Waste management - cytokines, growth factors and cachexia.. Cytokine Growth Factor Rev.

[25] Myers JS (2008). Proinflammatory cytokines and sickness behavior: implications for depression and cancer-related symptoms.. Oncol Nurs Forum.

[26] Reyes-Gibby CC, Wu X, Spitz M, Kurzrock R, Fisch M (2008). Molecular epidemiology, cancer-related symptoms, and cytokines pathway.. Lancet Oncol.

[27] Seruga B, Zhang H, Bernstein LJ, Tannock IF (2008). Cytokines and their relationship to the symptoms and outcome of cancer.. Nat Rev Cancer.

[28] Wood LJ, Nail LM, Gilster A, Winters KA, Elsea CR (2006). Cancer chemotherapy-related symptoms: evidence to suggest a role for proinflammatory cytokines.. Oncol Nurs Forum.

[29] Wheler J, Tsimberidou AM, Hong D, Naing A, Jackson T (2009). Survival of patients in a Phase 1 Clinic: the M. D. Anderson Cancer Center experience.. Cancer.

[30] Cleeland CS, Mendoza TR, Wang XS, Chou C, Harle MT (2000). Assessing symptom distress in cancer patients: the M.D. Anderson Symptom Inventory.. Cancer.

[31] Quinn J, Kramer N, McDermott D (2008). Validation of the Social Security Death Index (SSDI): An Important Readily-Available Outcomes Database for Researchers.. West J Emerg Med.

[32] Social Security Death Index Access Webpage through http://www.genealogybank.com/gbnk/ssdi/. Accessed March 2010

[33] Billewicz WZ, Kemsley WF, Thomson AM (1962). Indices of adiposity.. Br J Prev Soc Med.

[34] Mourtzakis M, Prado CM, Lieffers JR, Reiman T, McCargar LJ (2008). A practical and precise approach to quantification of body composition in cancer patients using computed tomography images acquired during routine care.. Appl Physiol Nutr Metab.

[35] Shen W, Punyanitya M, Wang Z, Gallagher D, St-Onge MP (2004). Total body skeletal muscle and adipose tissue volumes: estimation from a single abdominal cross-sectional image.. J Appl Physiol.

[36] Shen W, Punyanitya M, Wang Z, Gallagher D, St-Onge MP (2004). Visceral adipose tissue: relations between single-slice areas and total volume.. Am J Clin Nutr.

[37] Mitsiopoulos N, Baumgartner RN, Heymsfield SB, Lyons W, Gallagher D (1998). Cadaver validation of skeletal muscle measurement by magnetic resonance imaging and computerized tomography.. J Appl Physiol.

[38] Heymsfield SB, Smith R, Aulet M, Bensen B, Lichtman S (1990). Appendicular skeletal muscle mass: measurement by dual-photon absorptiometry.. Am J Clin Nutr.

[39] Miller KD, Jones E, Yanovski JA, Shankar R, Feuerstein I (1998). Visceral abdominal-fat accumulation associated with use of indinavir.. Lancet.

[40] Kurzrock R, Benjamin RS (2005). Risks and benefits of phase 1 oncology trials, revisited.. N Engl J Med.

[41] Horstmann E, McCabe MS, Grochow L, Yamamoto S, Rubinstein L (2005). Risks and benefits of phase 1 oncology trials, 1991 through 2002.. N Engl J Med.

[42] Finlay E, Lu HL, Henderson H, O'Dwyer PJ, Casarett DJ (2009). Do phase 1 patients have greater needs for palliative care compared with other cancer patients?. Cancer.

[43] Italiano A, Massard C, Bahleda R, Vataire AL, Deutsch E (2008). Treatment outcome and survival in participants of phase I oncology trials carried out from 2003 to 2006 at Institut Gustave Roussy.. Ann Oncol.

[44] Arkenau HT, Barriuso J, Olmos D, Ang JE, de Bono J (2009). Prospective validation of a prognostic score to improve patient selection for oncology phase I trials.. J Clin Oncol.

[45] Garrido-Laguna I, Janku F, Vaklavas C, Falchook GS, Fu S (2011). Validation of the royal marsden hospital prognostic score in patients treated in the phase I clinical trials program at the MD Anderson Cancer Center.. Cancer.

[46] Antoun S, Birdsell L, Sawyer MB, Venner P, Escudier B (2010). Association of skeletal muscle wasting with treatment with sorafenib in patients with advanced renal cell carcinoma: results from a placebo-controlled study.. J Clin Oncol.

[47] Baumgartner RN, Koehler KM, Gallagher D, Romero L, Heymsfield SB (1998). Epidemiology of sarcopenia among the elderly in New Mexico.. Am J Epidemiol.

[48] Kamel HK (2003). Sarcopenia and aging.. Nutr Rev.

[49] Evans W (1997). Functional and metabolic consequences of sarcopenia.. J Nutr.

[50] Shaffer JR, Kammerer CM, Reich D, McDonald G, Patterson N (2007). Genetic markers for ancestry are correlated with body composition traits in older African Americans.. Osteoporos Int.

[51] Uretsky S, Messerli FH, Bangalore S, Champion A, Cooper-Dehoff RM (2007). Obesity paradox in patients with hypertension and coronary artery disease.. Am J Med.

[52] Kalantar-Zadeh K, Kopple JD (2006). Obesity paradox in patients on maintenance dialysis.. Contrib Nephrol.

[53] Schmidt D, Salahudeen A (2007). The obesity-survival paradox in hemodialysis patients: why do overweight hemodialysis patients live longer?. Nutr Clin Pract.

